# A Step Forward in the Conceptualization and Measurement of Parental Burnout: The Parental Burnout Assessment (PBA)

**DOI:** 10.3389/fpsyg.2018.00758

**Published:** 2018-06-06

**Authors:** Isabelle Roskam, Maria-Elena Brianda, Moïra Mikolajczak

**Affiliations:** Psychological Sciences Research Institute, Université catholique de Louvain, Louvain-la-Neuve, Belgium

**Keywords:** parent, burnout, exhaustion, questionnaire, test, psychometrics

## Abstract

So far, the conceptualization and measurement of parental burnout have been deduced from those of job burnout. As a result, it is unclear whether current measures of parental burnout constitute the best representation of the parental burnout construct/syndrome: the possibility cannot be excluded that some dimensions ought to be added, which would change the structure and definition of parental burnout. In this study, the conceptualization and measurement of parental burnout were approached using an inductive method, in which the parental burnout phenomenon was (re)constructed based solely on the testimonies of burned-out parents. Items extracted from their testimonies were presented to a sample of French-speaking and English-speaking parents (*N* = 901) and submitted to factor analyses. An identifiable parental burnout syndrome including four dimensions was found (exhaustion in one's parental role, contrast with previous parental self, feelings of being fed up with one's parental role and emotional distancing from one's children). The resulting instrument, the Parental Burnout Assessment (PBA) presents good validity. Factorial invariance across gender and languages was also found. Finally, the results of this study replicate previous findings that psychological traits of the parents, parenting factors, and family functioning account for more variance in parental burnout than sociodemographic factors.

## Introduction

In their 2014 article, “Is burnout solely job-related? A critical comment,” Bianchi et al. (2014) questioned the view of burnout as a work-related condition. They argued that because enduring chronic stress—the putative cause of burnout—is not limited to work, the burnout phenomenon cannot be confined to work. According to these authors, any activity that can elicit frequent and intense stress response could contribute to the development of burnout. This position, controversial at the time, nevertheless echoes that of Pines and Aronson (1988) for whom burnout “can occur in all spheres that give people a sense of meaning” (p. 208). Because parenting has been shown to be a both complex and stressful activity (Abidin, 1990; Crnic and Low, 2002; Deater-deckard, 2014) and because children give meaning to their parents' lives (ONS-UK, 2012), parenting should be a likely candidate to produce burnout—if burnout exists outside work.

In 2017, Roskam, Raes and Mikolajczak provided preliminary evidence in favor of the existence of parental burnout. They first adapted the items of the Maslach Burnout Inventory© (MBI, Maslach et al., 1986) so that all items referred unambiguously to the parental context and then entered the 22 original work-related items together with 22 new parenting-related items in an exploratory factor analysis (Roskam et al., 2017). The results showed that professional and parental items loaded on separate components (i.e., three for professional and three others for parental burnout). Because the “depersonalization” subscale was weaker in the parental context, the authors replaced this subscale with an emotional distancing subscale. The validation study that followed resulted in the Parental Burnout Inventory (PBI), a measure of parental burnout encompassing three factors: exhaustion in one's parental role, emotional distancing from one's children, and loss of parental efficacy and accomplishment.

This preliminary evidence in favor of the existence and specificity of parental burnout was soon followed by a second, crucial piece of evidence: parental burnout was found to predict outcomes that were not predicted by job burnout. While both forms of burnout equally predict somatic complaints, sleep disorders, and addictive behaviors, parental burnout has a unique effect on neglectful and violent behaviors toward children (Mikolajczak et al., 2018). The results hold even after controlling for social desirability and depression. Taken together, the results of these studies constitute arguments in favor of the existence of non-work-related burnouts and of parental burnout in particular. Yet, because the Parental Burnout Inventory was built from the Maslach Burnout Inventory©, it remains unclear whether the tridimensional structure that emerged from the first studies is the *best* representation of the parental burnout construct/syndrome. The possibility cannot be excluded that other dimensions ought to be added, which would change the structure and definition of parental burnout.

Therefore, the aim of the current study was to go deeper into the conceptualization and measurement of parental burnout using a totally different method. So far, studies have relied on a deductive approach (i.e., parental burnout items and dimensions were deduced from those of job burnout). An inductive approach was therefore used: we reconstructed the parental burnout phenomenon based solely on the experience of burned-out parents. Items were extracted from testimonies of burned-out parents, and were then presented to a large sample of parents and submitted to exploratory factor analysis. If the dimensions emerging from the inductive method had little in common with those deduced from the MBI, this would call into question the existence of parental burnout as a specific and identifiable syndrome. By contrast, if the dimensions resulting from the inductive method are close to those deduced from the MBI, this would provide additional evidence in favor of the existence of parental burnout. These possibilities did not preclude the emergence of additional dimensions that would make it possible to capture the experience of burn-out even better and refine the construct and its measurement.

If the foregoing step were to confirm the existence of an identifiable parental burnout syndrome, the instrument resulting from the inductive approach would then be expected to show internal validity, high convergence with the Parental Burnout Inventory (PBI) and a highly similar pattern of correlations with correlates. Based on the results of a large study of the correlates of parental burnout using the PBI (Le Vigouroux et al., 2017; Mikolajczak et al., 2017), both measures of parental burnout would be expected to show few if any correlations with demographic variables, but moderate to large correlations with coparenting disagreement, family disorganization, neuroticism. If these hypotheses were corroborated, the instrument stemming from the inductive approach would constitute a free alternative to the Parental Burnout Inventory.

## Methods

### Sample

Data were collected from a sample of 901 English-speaking (71.8%) and French-speaking (28.2%) parents. The sample comprised 79.57% women. Participants were aged 20 to 59 (mean age = 36.71; *SD* = 6.84). 53.38% parents came from England, 26.41% from Belgium, 9.65% from the United States. The remaining 10.56% were from France, Canada, Ireland, Wales, Scotland, and other European countries. The mean number of siblings was 2.10 (*SD* = 0.70), ranging from 1 to 7. Their children's ages ranged from 0 to 39 years, and 41% of the parents had at least one child younger than 5 years. Among the parents, 3.7% reported having one child suffering from a chronic or severe disease, 6.4% from a disability, and 16% from a behavioral, emotional, or learning disorder. The educational level of the parents was calculated as the number of years of education they had completed from first grade onward. Of the participants, 42.7% were educated to secondary level, 33.7% had a first degree from university or college, 23.6% a master's degree, a Ph.D., or MBA degree. With regard to their work arrangements, 31.4% worked part-time while 43.3% worked full time. The remaining 25.3% were unemployed, on unpaid leave, on parental leave, or working as housewives/househusbands (16%). Of the parents, 79% were living with the father/mother of their child(ren), 8.6% were living with a partner who was not the father/mother of their child(ren) (i.e., blended family), 3.3% were single parents because they chose to have and/or to raise their child(ren) without a partner (i.e., single parenthood by choice), and 9.1% were single parents following a divorce or the death of the partner (i.e., single parenthood by circumstance).

### Procedure

The current study was approved by the Institutional Review Board. Data were collected from English-speaking parents on the Prolific platform (https://www.prolific.ac/) while French-speaking parents were informed about the research program through social networks, websites, or by word of mouth. On the Prolific platform, parents were rewarded £3 for their participation. The French-speaking parents who completed the questionnaire had the opportunity to enter a lottery with a chance of winning €200. Participants who wished to participate in the lottery had to provide their email address, but the latter was disconnected from their questionnaire.

The participants completed an online survey which was presented as a study about “Parenting today/Ê*tre Parent Aujourd'hui*.” Parents were eligible to participate in the studies only if they had (at least) one child still living at home. The informed consent they signed allowed participants to withdraw at any stage without having to justify their withdrawal. They were also assured that data would remain anonymous. The questionnaire was completed with a forced choice option, ensuring a dataset with no missing data.

First of all, a potential measure of parental burnout was designed based on thorough testimonies from five French-speaking exhausted parents. These testimonies were gathered by two collaborators of ours who were interested in going deeper into the experience of burned-out parents through interpretative phenomenological analysis (see in the current Research Topic Hubert and Isabelle, 2018). Parents participated in their qualitative research voluntarily in response to an advertisement displayed on social networks, websites and forums of parents, in particular a blog dedicated to maternal exhaustion, i.e., epuisement-maternel.com; this meant that they were already applying the term “parental burnout/exhaustion” to their feelings. Recruitment was made by means of this advertisement: “*As part of a research study on parental burnout/exhaustion conducted in the Department of Psychology at the University of Louvain, we are looking for parents who are willing to give us their testimony during individual interviews, in order to get closer to their experience and perhaps gain a better understanding of parental exhaustion*.”

In the end, only mothers responded to this advertisement. Parents of children aged 0 to 18 months were excluded in order to avoid confusion with post-partum depression. The mothers interviewed were between 30 and 42 years old and had two children (between 2 and 14 years old). Four of the five mothers lived with their children's father. Two of these worked (one part-time and the other full-time), a third had chosen to stop working and a fourth was on sick leave. The fifth mother was separated from her partner and worked full-time.

The collection of parents' testimony was done through unstructured interviews which were recorded for the purpose of analysis. The interviews were conducted by a trained research assistant who met the mothers twice. The first interview lasted about 2 h. The second took place a few weeks later, after sending the transcript to the mother. It was aimed to ensure that the transcript matched what she wanted to say and reflected her experiences, and to allow her to provide extra detail or corrections where necessary. Interviews took the form of discussions favoring the sharing of an intimate story in connection with the parent's experience. Four mothers chose to be interviewed at home, when they were alone. One mother preferred to be interviewed elsewhere. Before each interview, an informed consent and an agreement concerning the recording and anonymous utilization of interview excerpts were signed by the mothers.

Based on the thematic analysis done by Hubert and Isabelle (2018), words, phrases, and sentences most representative of burned-out parents' feelings and thoughts were used to produce 52 items. These items aimed to reflect the regularities/communalities in burned-out parents' inner experience. The list was submitted to a double-blind translation procedure to provide both an English and a French version. Two items were removed before distributing the survey for ethical reasons, i.e., *I feel guilty about no longer having any desire to see my children; I think that my life might be better without my children*. A final list of 50 items was finally included in the survey. Items were rated on 7-point Likert scales: never (0), a few times a year or less (1), once a month or less (2), a few times a month (3), once a week (4), a few times a week (5), every day (6). Sociodemographic questions and validated measures of parental burnout (i.e., the PBI), neuroticism, coparenting, and family disorganization were added to the survey. An English version of the survey was provided to the English-speaking participants and a French version to the French-speaking participants. A measure of job burnout was also added to the survey distributed to English-speaking parents.

### Measures

#### Sociodemographic factors

Participants were asked about their age, gender, type of family (single parenthood by choice or by circumstance, living with the children's father/mother, or blended family), level of education, work regimen, number of children, and for each child: gender, age, and whether the child suffered from a disease, disability, or behavioral/emotional/learning disorder (yes-no). If the parent answered “yes” for at least one child, he or she was asked to fill in a short questionnaire about the impact of having a child with special needs on his or her own life (see Gérain and Zech, 2018).

#### Parental burnout

Parental burnout was assessed for comparative purposes with the Parental Burnout Inventory^1^ (PBI, Roskam et al., 2017), a 22-item self-report questionnaire which has been created based on a deductive approach starting from the tridimensional model of professional burnout (Maslach and Jackson, 1981; Maslach et al., 2001). The PBI consists of three subscales: Emotional Exhaustion (8 items) (e.g., I feel emotionally drained by my parental role), Emotional Distancing (8 items) (e.g., I sometimes feel as though I am taking care of my children on autopilot), and Loss of Parental Accomplishment (6 items) [e.g., I accomplish many worthwhile things as a parent (reversed)]. Items are rated on 7-point Likert scales: never (0), a few times a year or less (1), once a month or less (2), a few times a month (3), once a week (4), a few times a week (5), every day (6). In the current sample, Cronbach's alphas were 0.92, 0.89, 0.85 for the three subscales and 0.91 for the global score (i.e., the sum score of all PBI items). Alphas were similar in the French and English versions of the questionnaire with respectively 0.94 and 0.92 for Emotional Exhaustion, 0.87 and 0.90 for Emotional Distancing, 0.85 and 0.85 for Loss of Parental Accomplishment, and 0.92 and 0.91 for the global score.

#### Neuroticism

Neuroticism was assessed with the Neuroticism subscale of the Big Five Inventory (BFI; John et al., 1991; Plaisant et al., 2010). The Neuroticism subscale includes 8 items rated on a five-point Likert-type scale from 1 (strongly disagree) to 5 (strongly agree). Example of items are “is temperamental, gets emotional easily” or “is emotionally stable, not easily upset” (Reversed). In order to minimize shared variance with the current state of the parent, the instructions asked the parent to indicate whether each description reflected what they were like in general (i.e., their nature, rather than how they had felt over the last few weeks or months). In the current sample, Cronbach's alpha was 0.85 in the current sample, and 0.83 and 0.87 in the French and English versions of the questionnaire respectively.

#### Coparenting disagreement

Coparenting disagreement was assessed by means of the Agreement subscale of the revised Co-Parenting Scale (CPS, Feinberg et al., 2012), which consists of 4 items [e.g., “My partner and I have the same goals for our child(ren)”]. Items are rated on a seven-point Likert-scale from 1 (not at all true for us) to 7 (absolutely true for us). The items were reversed so that higher scores meant coparenting disagreement. Cronbach's alpha was 0.82 in the current sample, and 0.75 and 0.84 in the French and English versions of the questionnaire respectively.

#### Family disorganization

Family disorganization was assessed with the CHAOS (Confusion Hubbub And Order Scale), a 15-item measure of “environmental confusion and disorganization in the family,” i.e., high levels of noise, crowding, and home traffic, in children's development (Matheny et al., 1995). Example of items are: “We can usually find things when we need them” or “The atmosphere in our home is calm.” Based on current usage, a single score was derived from the CHAOS questionnaire to represent the parent's report of home characteristics, corresponding to the simple sum of responses for the 15 items. The true or false responses were scored so that a higher score represented more chaotic, disorganized, and time-pressured homes. In the current study, reliability was 0.80, 0.75, and 0.82 in the French and English versions of the questionnaire respectively.

#### Job burnout

Job burnout was assessed with the Maslach Burnout Inventory-General Survey (MBI-GS; Schaufeliet et al., 1996). The MBI is a widely used 16-item questionnaire encompassing three factors: emotional exhaustion (5 items), cynicism (5 items), and professional efficacy (6 items). Items are in the form of “I feel emotionally drained from my work.” The instruction is as follows: “Please read each statement carefully and decide if you ever feel this way about your job.” Likert-type scales are in the form of “How often?”, with a 7-point scale of frequency, i.e., never (0), a few times a year or less (1), once a month or less (2), a few times a month (3), once a week (4), a few times a week (5), every day (6). The global score is computed after reversing the items of the professional efficacy factor, so that higher scores indicate greater burnout. In the current sample, reliability was 0.82.

### Data analyses

We started by factor-analyzing the 50 items reflecting burned-out parents' experience. We excluded unsatisfactory items and then analyzed the internal validity of the resulting instrument (hereafter named Parental Burnout Assessment; PBA). Afterwards, we examined its convergence with the Parental Burnout Inventory (PBI) and compared the two instruments' pattern of correlations with other variables (demographic variables, coparenting disagreement, family disorganization and neuroticism, and job burnout).

For the purpose of factor analyses, the sample was split into two subsamples of 450 and 451 participants respectively in order to compute Exploratory Factor Analysis (EFA) and Confirmatory Factor Analysis (CFA) on two different samples. The 901 subjects were randomly assigned to one of the two subsamples. The comparability of the two subsamples was checked with crosstabs and χ^2^ analyses for categorical variables (e.g., parent gender) and with one-way ANOVAs for continuous variables (e.g., parent age). They were found to be strictly similar with regard to socio-demographic characteristics. Further analyses were conducted on the entire sample (*N* = 901). Statistical analyses were all computed using SPSS 25 (IBM, 2017) except for CFA, which was computed using Stata 16 software (StataCorp., 2016).

The 50 initial items were subjected to an EFA (using maximum likelihood estimation with Varimax rotation) computed on the first subsample (*N* = 451). EFA permitted us to explore the number of meaningful underlying dimensions and to retain a pool of items. We applied parallel analysis to our dataset, a method that is currently considered the most reliable procedure to determine the correct number of factors (Hayton et al., 2004). These analyses were based on a comparison between eigenvalues from a factor analysis of the actual data and eigenvalues from a factor analysis of a random dataset. We obtained the eigenvalues and standard deviations generated from completely random data (and necessary to perform parallel analysis) through the “Marley Watkins Monte Carlo PCA for Parallel Analysis” program (Watkins, 2002a) using the following parameters: 50 variables, 451 participants, and 1,000 replications. The number of components to be retained was based on the number of actual data eigenvalues higher than the upper 95% confidence limit of random data eigenvalues.

A CFA was then performed on the second subsample (*N* = 450). The measurement model included four latent variables representing the concepts of exhaustion, contrast with previous parental self, feelings of being fed up and emotional distancing, and their indicators consisting of 9 items for exhaustion, 6 for contrast with previous parental self, 5 for feelings of being fed up, and 3 for emotional distancing^2^. Analyses were conducted using the maximum likelihood estimation. Several goodness-of-fit indices were used to determine the acceptability of the models. In addition to the chi-square model, which is highly sensitive to sample size and leads to model rejection even when the model misspecification is relatively minor (Hayduk, 1996; Byrne, 1998), the Root Mean Square Error of Approximation (RMSEA), the standardized root mean square residual (SRMS), the comparative fit index (CFI), and the Tucker-Lewis index (TLI) were used (Acock, 2013). For CFI and TLI, values close to 0.90 or greater are acceptable to good. RMSEA and SRMR should preferably be less than or equal to 0.08 (Hu and Bentler, 2009). Reliability was estimated with Cronbach's alpha coefficients (α).

We compared the French-speaking and English-speaking parents' factor structures, and mothers' and fathers' factor structures, using the Coefficient of Congruence Program of Watkins (2002b). The congruence coefficient (rc) is an index of factor similarity. It is typically used to determine the factorial invariance of solutions across samples or studies. The results were interpreted following the threshold points proposed by Lorenzo-Seva and Ferrando (2006). Values equal to or higher than 0.95 reveal that the two factors compared may be considered as equal, values between 0.85 and 0.94 indicate that they display fair similarity, and values lower than 0.85 that they do not display any similarity at all.

For convergent validity, we first computed correlations (Pearson and Kendall) between the sum scores of the PBA and the PBI, and between the mean scores of their subscales. Second, we appraised the frequency of parents in five categorical levels (corresponding to the levels of the response scale) according to whether they displayed at least 2/3 of the symptoms (66.6%) never to a few times a year (category 1), once a month or less (category 2), a few times a month (category 3), a few times a week (category 4), or every day (category 5). For cross-validation purposes, this criterion was applied both in the PBI and in the PBA scores; we then compared the percentage of parents in the five burnout levels according to the two instruments. The frequencies were also computed across samples (French-speaking vs. English-speaking) and gender (mothers vs. fathers). To appraise the probability of being categorized in the highest category with the PBI according to the PBA category levels, a binary score, i.e., being in the highest category or not according to the PBI, was entered in a binary logistic regression as the dependent variable with the five category levels of the PBA as the predictor.

With regard to the relation between the PBA and other variables, we computed correlations between the sum score of the PBA and the mean scores of the ordinal/continuous variables, i.e., age, educational level, number of children, neuroticism, coparenting disagreement, family disorganization, and job burnout, aiming at replicating previously found associations with the PBI (Mikolajczak et al., 2017). To take the convergent validation process a step further, we compared the correlations found for the PBA and the PBI with these other variables. ANOVAs were computed to test the effect of categorical sociodemographic factors on PBA scores i.e., gender, family type (single parenthood by choice or by circumstance, living with the children's father/mother, or blended family), working time (part-time vs. full-time), having at least one child with special needs and having at least one child younger than 5 years old.

## Results

### Factor analyses

#### Exploratory factor analysis of the 50 items

Parallel analyses conducted on the 50 items suggested a four-factor structure. The first five eigenvalues from the actual data were 24.63, 3.04, 2.07, 1.61, and 1.39; the corresponding 95th percentile random data eigenvalues were 1.73, 1.65, 1.60, 1.56, 1.52, and 1.48. The four factors displayed in the EFA explained 54.48% of the variance. The first four dimensions were found to be meaningful, with the first one consisting mainly of feelings of being fed up, the second one of contrast with previous parental self, and the third and fourth ones of a mix of exhaustion feelings in parental role. The items and loadings of the EFA are presented in Supplemental Material Table S1. We then removed items with evident cross-loadings across three factors or more (>0.30; e.g., *I feel frustrated in my role as a parent*), as well as items with the highest loading on the fifth or sixth factor (e.g., *I only half-listen to what my children tell me*). In case of redundancy, we removed one of the two items (e.g., *Thinking of everything I have to do as a mum/dad makes me feel like staying in bed*, and *I find it exhausting just thinking of everything I have to do for my children*). We also removed items whose meaning could be interpreted outside the scope of parental burnout (e.g., *My children are a source of anxiety*). This resulted in a list of 23 items that we subjected to another EFA. The four-factor structure accounted for 66.59% of the variance. Based on items' meaning, the first dimension was labeled “exhaustion in one's parental role,” the second one “contrast with previous parental self,” the third one “feelings of being fed up,” and the fourth one “emotional distancing from one's children.” The items, loadings, and reliability estimates of the four-factor structure of the PBA are presented in Table 1. The 23-item version of the PBA was then subjected to a CFA on the second subsample.

**Table 1:** Loading parameter estimates in EFA from the four-factor solution and reliability estimates for the 23-item version of the PBA in subsample 1 (*N* = 451) and standardized regression weights from CFA and reliability estimates for the final 23-item version of the PBA in subsample 2 (*N* = 450).

		**EFA**				**CFA**			
		**EP**	**SD**	**LA**	**ED**	**EP**	**SD**	**LA**	**ED**
EX1	I feel completely run down by my role as a parent	**0.824**	0.254	0.291	−0.061	0.84			
EX2	I have the sense that I'm really worn out as a parent	**0.780**	0.322	0.176	0.119	0.86			
EX3	I'm so tired out by my role as a parent that sleeping doesn't seem like enough	**0.726**	0.131	0.068	−0.031	0.70			
EX4	When I get up in the morning and have to face another day with my child(ren), I feel exhausted before I've even started	**0.718**	0.226	0.206	0.287	0.82			
EX5	I find it exhausting just thinking of everything I have to do for my child(ren)	**0.656**	0.207	0.165	0.317	0.75			
EX6	I have zero energy for looking after my child(ren)	**0.656**	0.334	0.333	0.091	0.80			
EX7	My role as a parent uses up all my resources	**0.640**	0.185	0.277	0.316	0.80			
EX8	I sometimes have the impression that I'm looking after my child(ren) on autopilot	**0.552**	0.311	0.165	0.348	0.71			
EX9	I'm in survival mode in my role as a parent	**0.541**	0.276	0.371	0.295	0.73			
CO1	I don't think I'm the good father/mother that I used to be to my child(ren)	0.323	**0.764**	0.212	0.050		0.83		
CO2	I tell myself that I'm no longer the parent I used to be	0.279	**0.755**	0.259	0.237		0.85		
CO3	I'm ashamed of the parent that I've become	0.240	**0.708**	0.306	0.218		0.88		
CO4	I'm no longer proud of myself as a parent	0.262	**0.699**	0.257	0.282		0.88		
CO5	I have the impression that I'm not myself any more when I'm interacting with my child(ren)	0.289	**0.681**	0.298	0.289		0.83		
CO6	I feel as though I've lost my direction as a dad/mum	**0.450**	**0.628**	0.307	0.014		0.78		
FU1	I can't stand my role as father/mother any more	0.186	0.172	**0.824**	0.129			0.81	
FU2	I can't take being a parent any more	0.190	0.268	**0.747**	0.223			0.83	
FU3	I feel like I can't take any more as a parent	0.292	0.289	**0.689**	0.134			0.83	
FU4	I feel like I can't cope as a parent	**0.423**	**0.403**	**0.636**	0.074			0.86	
FU5	I don't enjoy being with my child(ren)	0.231	0.312	**0.560**	0.276			0.75	
ED1	I do what I'm supposed to do for my child(ren), but nothing more	0.233	0.306	0.364	**0.542**				0.69
ED2	Outside the usual routines (lifts in the car, bedtime, meals), I'm no longer able to make an effort for my child(ren)	0.258	0.368	**0.462**	**0.505**				0.84
ED3	I'm no longer able to show my child(ren) how much I love them	0.076	0.385	0.379	**0.412**				0.72
	α	0.93	0.93	0.90	0.81	0.93	0.94	0.91	0.77

*Factor loadings in EFA > |0.40| are in bold; EX, Exhaustion in Parental role; CO, Contrast in parental self; FU, Feelings of being fed up; ED, Emotional Distancing*.

### Confirmatory factor analysis and reliability of the PBA

All the estimated factor loadings found in the CFA were significant at *p* < 0.001. Standardized factor loadings ranged between 0.69 and 0.88, and reliability estimates were high. Standardized factor loadings are displayed in Table 1. Correlations between the four factors were 0.76 (exhaustion-contrast with previous parental self), 0.76 (exhaustion-feelings of being fed up), 0.66 (exhaustion-emotional distancing), 0.78 (contrast with previous parental self-feelings of being fed up), 0.76 (contrast with previous parental self-emotional distancing), and 0.79 (feelings of being fed up-emotional distancing). With regard to fit indices, $\chi_{\left(193\right)}^{2}$ = 685.71 was significant at *p* = 0.001, indicating that there is some discrepancy between the hypothesized model and the data. Other fit measures demonstrated a very good fit to the data, with CFI = 0.94, TLI = 0.93, RMSEA = 0.07, and SRMR = 0.04. These results confirm the validity of the four-factor internal structure of the PBA.

### Comparison of the factor structure of french-speaking and english-speaking parents

Congruence coefficients obtained when comparing the factorial structure of French-speaking parents with that of English-speaking parents were 0.98 for factor 1 (exhaustion in parental role), 0.96 for factor 2 (contrast with previous parental self), 0.94 for factor 3 (feelings of being fed up), and 0.95 for factor 4 (emotional distancing). The loadings of the four factors in the two subsamples are presented in Supplemental Material Table S2. Based on Lorenzo-Seva and Ferrando (2006) threshold points, these results suggest that the four factors compared in the two samples may be considered as similar, suggesting invariance of the factor structure of the PBA across samples.

### Comparison of the factor structure of mothers and fathers

Congruence coefficients obtained when comparing the factorial structure of mothers with that of fathers were 0.98 for factor 1 (exhaustion), 0.89 for factor 2 (contrast with previous parental self), 0.88 for factor 3 (feelings of being fed up), and 0.88 for factor 4 (emotional distancing). The loadings of the four factors in the two subsamples are presented in Supplemental Material Table S2. These results suggest that the *exhaustion* factor is equal across samples while the three other factors display fair similarity.

Based on the results found for the internal structure of the PBA and in order to investigate both the convergent validity and the relations between PBA and other variables, scores were computed for the four validated PBA factors. These were obtained by summing the item scores in each of the four subscales; the higher the scores, the higher the burnout. A global score was also computed, which was found to be highly reliable in the pooled sample of 901 parents (α = 0.96). Descriptive statistics of the PBA scores in the pooled sample are presented in Table 2.

**Table 2:** Descriptive statistics of PBA and PBI subscales and global score in the pooled sample and according to gender, sample, family type, working time, having a child with special needs and having at least one child younger than 5 years old.

	**Pooled sample (*N* = 901)**	**Gender**		**Sample**		**Family type**				**Working time**		**Having a child with special needs**		**Having at least one child younger than 5 years old**	
		**Mothers (*N* = 717)**	**Fathers *(N* = 184)**	**French-speaking (*N* = 254)**	**English-speaking (*N* = 647)**	**Single choice (*N* = 29)**	**Single burdened (*N* = 88)**	**Blended (*N* = 85)**	**Two-Parent (*N* = 699)**	**Part-time (*N* = 283)**	**Full-time (*N* = 390)**	**No (*N* = 726)**	**Yes (*N* = 175)**	**No (*N* = 531)**	**Yes (*N* = 370)**
	***M (SD)***	***M (SD)***	***M (SD)***	***M (SD)***	***M (SD)***	***M (SD)***	***M (SD)***	***M (SD)***	***M (SD)***	***M (SD)***	***M (SD)***	***M (SD)***	***M (SD)***	***M (SD)***	***M (SD)***
**PBA**															
EX (9 items)	15.08 (12.50)	15.91 (12.87)	11.84 (10.35)	13.55 (10.87)	15.68 (13.05)	13.65 (13.12)	16.02 (13.97)	16.20 (13.11)	14.88 (12.22)	14.95 (12.69)	13.12 (11.26)	14.14 (11.90)	18.96 (14.12)	12.22 (11.29)	17.07 (12.92)
CO (6 items)	5.80 (7.60)	6.17 (7.98)	4.34 (5.64)	4.92 (6.89)	6.14 (7.84)	5.58 (8.02)	6.72 (8.54)	7.95 (8.55)	5.43 (7.29)	5.85 (7.78)	4.35 (5.85)	5.28 (7.17)	7.94 (8.86)	5.37 (6.77)	6.10 (8.12)
FU (5 items)	3.27 (5.03)	3.50 (5.19)	2.36 (4.23)	4.60 (5.68)	2.74 (4.65)	1.96 (2.92)	3.82 (5.98)	4.02 (4.89)	3.16 (4.97)	3.74 (5.80)	2.54 (3.99)	2.89 (4.54)	4.84 (6.45)	2.75 (4.47)	3.63 (5.36)
ED (3 items)	1.90 (2.97)	1.92 (3.00)	1.82 (2.84)	2.58 (3.09)	1.63 (2.88)	2.37 (3.18)	2.29 (3.49)	2.27 (3.14)	1.78 (2.86)	1.95 (3.05)	1.65 (2.59)	1.74 (2.73)	2.56 (3.75)	1.84 (2.83)	1.94 (3.06)
**Global score** **(23 items)**	26.05 (24.96)	**27.51** **(26.01)**	**20.34** **(19.36)**	25.67 (24.22)	26.21 (25.25)	23.58 (22.27)	28.87 (29.53)	**30.44** **(26.41)**	**25.27** **(24.21)**	**26.49** **(26.30)**	**21.68** **(20.63)**	**24.07** **(23.17)**	**34.30** **(30.00)**	**22.19** **(22.46)**	**28.75** **(26.24)**
Statistics		*F*_(1, 899)_ = 12.12^***^						*F*_(1, 782)_ = 3.38^†^		*F*_(1, 671)_ = 7.06^**^		*F*_(1, 899)_ = 24.29^***^		*F*_(1, 899)_ = 15.29^***^	
**PBI**															
EE (8 items)	13.75 (11.44)	14.08 (11.78)	12.47 (9.94)	10.67 (10.19)	14.92 (11.68)	13.00 (12.31)	14.81 (13.24)	14.54 (11.45)	13.54 (11.17)	13.00 (11.37)	12.13 (10.72)	12.79 (10.87)	17.69 (12.83)	11.27 (10.65)	15.48 (11.66)
ED (8 items)	5.90 (7.46)	5.82 (7.28)	6.20 (8.11)	6.87 (6.97)	5.53 (7.61)	7.55 (9.25)	6.20 (7.52)	7.83 (8.14)	5.56 (7.25)	5.78 (6.91)	5.77 (7.45)	5.41 (6.81)	7.91 (9.45)	6.39 (7.86)	5.55 (7.15)
PA (6 items)	7.29 (6.66)	7.05 (6.48)	8.21 (7.25)	6.84 (5.81)	7.46 (6.95)	7.93 (8.59)	8.72 (8.28)	8.89 (7.16)	6.89 (6.22)	6.62 (6.12)	7.32 (6.41)	7.04 (6.59)	8.34 (6.83)	8.11 (6.86)	6.71 (6.45)
**Global score** **(22 items)**	26.94 (19.99)	26.96 (20.23)	26.83 (19.09)	24.39 (18.27)	27.91 (20.53)	28.48 (21.64)	29.75 (23.47)	**31.27** **(20.27)**	**26.00** **(19.33)**	25.41 (19.24)	25.23 (19.31)	**25.25** **(18.77)**	**33.95** **(23.17)**	25.78 (20.36)	27.76 (19.71)
Statistics								*F*_(1, 773)_ = 5.51^*^				*F*_(1, 899)_ = 27.33^***^			

* *p = 0.10*,

† *p = 0.05*,

** p = 0.01, and

*** *p = 0.001*.

*When comparing numbers for the PBI and the PBI, readers should keep in mind that the number of items differs between the PBA and the PBI (for the global score and for the factors). Statistical differences according to gender, sample, family type, working time, and having a child with special needs, are in bold. The results of ANOVAs are given for significant differences only. EX, Exhaustion in parental role; CO, Contrast in parental self; FU, Feelings of being fed up; ED, Emotional; EE, Emotional Exhaustion; PA, (Decreased) Personal Accomplishment*.

### Convergent validity

The correlations between the four subscales of the PBA and the three subscales of the PBI as well as the bivariate associations encompassing the two total scores are presented in Table 3. Coefficients between the two exhaustion factors were high, *r* = 0.86 and *tau* = 0.67, and the same was true for emotional distancing, *r* = 0.80 and *tau* = 0.60, and for the global scores, *r* = 0.84 and *tau* = 0.64, giving support to the good convergent validity of the PBA. The feelings of being fed up and the contrast with previous parental self-factors were moderately correlated to the three PBI dimensions, suggesting that they constitute dimensions specifically drawn from the inductive method under consideration in this paper which had not been fully identified by the deductive method inspired from the job burnout framework.

**Table 3:** Pearson and Kendall correlations between PBA and PBI (factors and global scores).

	**PBA**									
	**Exhaustion in parental role**		**Contrast in parental self**		**Feelings of being fed up**		**Emotional distancing**		**Total score**	
**PBI**	**Pearson**	**Kendall**	**Pearson**	**Kendall**	**Pearson**	**Kendall**	**Pearson**	**Kendall**	**Pearson**	**Kendall**
Emotional Exhaustion	0.86	0.67	0.68	0.50	0.67	0.49	0.56	0.38	0.84	0.66
Emotional Distancing	0.56	0.43	0.63	0.45	0.63	0.51	0.80	0.60	0.70	0.51
(Decreased) Personal Accomplishment	0.19	0.19	0.34	0.33	0.23	0.25	0.27	0.28	0.28	0.26
Total score	0.76	0.57	0.74	0.54	0.70	0.52	0.71	0.49	0.84	0.64

*All the correlations are significant at p < 0.001*.

Parents displaying at least 65% of the burnout symptoms (i.e., items) every day were considered to be in burnout. Following this rule, we examined whether the percentage of burned-out parents based on the PBA and the PBI was equal. Based on frequencies, 5.9% of the parents were found to be in burnout with the PBA against 5.3% for the PBI. The frequencies at each of the five burnout levels are shown in Table 4 according to the PBI and the PBA, across French- and English-speaking parents, and across gender. The distribution appears to be highly similar for the PBI and the PBA over the five burnout levels in the pooled sample, except between low and moderate risks. With regard to gender, the PBA seemed to discriminate between burned-out mothers and fathers to a higher extent than the PBI in the last two categories, i.e., high risk and burnout. The percentage of mothers in burnout or at high risk according to the PBA was higher than the percentage of fathers in the same situations. For the sample, the percentage of burned-out parents seemed to be slightly higher among English-speaking than French-speaking parents, suggesting some cross-cultural variability in the prevalence of parental burnout.

**Table 4:** Percentage of parents belonging to each category (higher categories = higher burnout scores) according to the PBA and the PBI.

**Burnout levels**	**Pooled sample (*****N*** = **901)**		**Mothers (*****N*** = **717)**		**Fathers (*****N*** = **184)**		**French-speaking (*****N*** = **254)**		**English-speaking (*****N*** = **647)**	
	**PBA**	**PBI**	**PBA**	**PBI**	**PBA**	**PBI**	**PBA**	**PBI**	**PBA**	**PBI**
Category 1	70.3	61.0	68.9	61.0	75.5	60.9	72.0	65.9	69.6	59.1
Category 2	12.4	16.7	11.7	17.5	15.2	13.6	12.2	16.7	12.5	16.7
Category 3	6.4	12.1	6.6	11.2	6.0	15.8	5.9	9.8	6.6	13.0
Category 4	5.0	4.9	5.9	4.8	1.6	5.4	5.1	5.3	4.9	4.8
Category 5	5.9	5.3	7.0	5.5	1.6	4.3	4.7	2.4	6.3	6.3

*Category 1, 2/3 of the symptoms never to a few times a year; Category 2, once a month or less; Category 3, a few times a month; Category 4, a few times a week; Category 5, every day*.

The results of the binary logistic regression showed that the odds of being in the highest burnout category rather than not being in this category with the PBI significantly increased as parents moved from one category level to the next with the PBA (odds ratio = 3.48). The model had a good pseudo-R^2^ (Nagelkerke's ${\text{R}}_{\text{N}}^{2}$ = 0.44) and the classification table indicated that it correctly predicted the outcome in 94.7% of cases.

### Relations with other variables

Correlation coefficients between the global score for parental burnout (measured either with the PBA or the PBI as a further means of assessing convergent validity), age, educational level, number of children, neuroticism, coparenting disagreement, family disorganization, and job burnout are presented in Table 5. Coefficients found for PBA were very close to those found for PBI, giving additional support to the good convergent validity of the PBA. None of the coefficients was statistically different from its counterpart in the other instrument. We also replicated previously found low associations between parental burnout and sociodemographic factors but moderate to high associations between parental burnout and neuroticism, coparenting disagreement, family disorganization, and job burnout (Mikolajczak et al., 2017; Roskam et al., 2017).

**Table 5:** Correlations between the PBA, the PBI and other variables under study.

	**PBA**	**PBI**
Age	0.07^*^	0.01
Educational level	0.01	0.01
Number of children	0.14^***^	0.10^**^
Neuroticism	0.47^***^	0.47^***^
Coparenting disagreement	0.22^***^	0.25^***^
Family disorganization	0.53^***^	0.57^***^
Job burnout	0.42^***^	0.48^***^

* *p < 0.05*,

** p < 0.01, and

*** *p < 0.001*.

The results of ANOVAs showed that the intensity of mothers' burnout assessed with the PBA was significantly higher than that of fathers. However, this difference according to gender was not displayed with the PBI. With regard to family type, there was no difference in burnout between single parents by choice and single parents by circumstance, either with the PBA or with the PBI. Also, burnout among parents in two-parent families did not differ from burnout in single parents by circumstance either with the PBA or with the PBI. Burnout among parents in two-parent families did not differ from burnout in single parents by choice either with the PBA or with the PBI. A last comparison for family type was made between parents in blended families and in two-parent families. These tended to differ from each other according to the PBA and differed significantly according to the PBI.

In terms of working time, parents working part-time displayed higher levels of burnout than parents working full-time with the PBA but not with the PBI. Parents having at least one child with special needs displayed higher levels of burnout than other parents with both the PBA and the PBI. Finally, having at least one child younger than 5 years old was associated with higher burnout with the PBA but not with the PBI. Descriptive statistics are given in Table 2. Overall, slightly more significant differences emerge with the PBA than with the PBI.

## Discussion

This study aimed to go deeper in the conceptualization and measurement of parental burnout using a radically different approach. In the first generation of studies, parental burnout was not conceptualized as a distinct syndrome from job burnout and, therefore, parents' burnout was measured via instruments that did not discriminate between the two forms of burnout (Lindhal-Norberg, 2007; Norberg, 2010; Lindström et al., 2011; Lindhal Norberg et al., 2014). In the second generation of studies, parental burnout conceptualization and measurement were *deduced from* those of job burnout (e.g., Roskam et al., 2017; Lebert-Charron et al., 2018). In the current study, which can be seen as instituting the third generation of studies, parental burnout and its measurement were approached using an *inductive method*, viz. starting from burned-out parents' testimonies.

The results of this study show substantial consistency between the dimensions emerging from the deductive and inductive approaches (resulting in the PBI and the PBA respectively). The first and most important dimension of the PBA, *Exhaustion in one's parental role*, is the same as in the PBI. The second dimension, i.e., *Contrast*, is not formally measured in the PBI (nor in the MBI) but is nonetheless inherent to the notion of burnout: if the current state of the person does not contrast with a previous period, the person cannot be said to be in burnout. Thus, the PBA materializes a very important diagnosis criterion of burnout that is not captured by any other burnout measure, although it should be. The third dimension of the PBA, “*Feelings of being fed up,”* differs somewhat from the “*Loss of parental accomplishment and efficacy*” of the PBI and suggests that, while all burned-out parents lose pleasure and fulfillment in their parental role, not all of them lose their efficacy. Finally, the last dimension, *Emotional distancing from one's children*, is the same in the PBA and the PBI.

In addition to the congruence between the *constructs* emerging from the deductive and inductive approach, there was also remarkable consistency between the correlations of the PBA and PBI with other variables (sociodemographics, neuroticism, coparental disagreement, family disorganization, etc.). A close comparison of the relationships of the PBA and PBI with these variables show that the correlation of one instrument with a given variable differed *at most* by 6% from the correlation of the other instrument with the same variable. This constitutes another argument in favor of the idea that the two instruments measure the same syndrome.

Taken together, the results of this study add to the growing body of evidence corroborating the view of Pines and Aronson (1988) and Bianchi et al. (2014) that the burnout phenomenon is not confined to work. However, the structure and content of parental burnout is somewhat different from job burnout from both theoretical and practical perspectives. As in Pelsma's work (1989) as well as in the validation study of the PBI (Roskam et al., 2017), depersonalization was found to be unsuitable in the parental context. Although highly exhausted employees may consider their clients or patients as numbers, highly exhausted parents cannot “dehumanize” their children. Even when they are at the end of their rope, parents cannot consider the flesh of their flesh as objects. This is an important difference between burnout in the professional and parental spheres. What they can do, however, is distance themselves from the source of exhaustion. In our clinical and research experience with parents of children with externalized disorders, i.e., conduct disorder or antisocial behavior, we have observed that exhausted parents disengage emotionally rather than physically, i.e., they provide practical care such as feeding or sleeping but became less emotionally involved, sensitive, and responsive to their offspring.

The results of the current study also support the validity of the PBA as a measure of parental burnout in both French-speaking and English-speaking parents. Finally, they replicate previous findings that psychological traits of the parents, parenting factors, and family functioning account for more variance in parental burnout than sociodemographic factors such as the number and age of children, working time, or family structure (Mikolajczak et al., 2017). With regard to this last point, the results of the current study shed some light on an intriguing result of Mikolajczak et al. (2017), who found no difference in average burnout score between single parents and parents with a spouse. In order to go deeper into this counterintuitive result, we differentiated here between single parents by choice and burdened single parents. Descriptive statistics showed that single parents by choice scored lower on burnout than parents with a partner, who themselves scored lower than burdened single parents. Although these differences were not statistically significant, this pattern of results fully explains why single parents (in general) did not score higher on burnout in previous studies.

The current study has the merit of providing an alternative to the measurement of parental burnout that can be considered, like the PBI, as a valid assessment method. High overlap between the two instruments and consistency regarding relations with other variables might be thought to suggest that the PBI and the PBA are commutable measures. However, this is not true, for at least four reasons. The first is that unlike the PBI, the PBA provides an important diagnostic criterion in the form of contrast with previous parental self. Although contrast is inherent to the notion of burnout, it is not formally assessed in the PBI. This diagnostic criterion is very important to make sure that the concept of burnout is employed to identify exhausted parents rather than permanently dismissive ones. The second reason for distinguishing the two instruments is that in the PBI (as in the MBI), the items of the Exhaustion and Emotional Distancing subscales evaluate burnout, i.e., the higher the score, the higher the burnout symptoms, while the items of the (Loss of) Parental Accomplishment and Efficacy subscale evaluate the inverse of burnout, i.e., the higher the score, the lower the burnout symptoms. However, the mere fact that a parent reports a limited level of accomplishment and efficacy does not mean that (s)he is in burnout. In the testimonies, burned-out parents reported that they did not enjoy being with their children anymore, not that they enjoyed being with their children slightly less. We strongly believe that items that directly assess burnout are preferable. In the PBA, all 23 items are formulated in a sense which measures burnout. The third reason is that the inductive method allowed us to nuance the (Loss of) Parental Accomplishment and Efficacy dimension. It shows that, in the case of parental burnout, the loss of pleasure and fulfillment in the parental role takes precedence over the loss of efficacy. The last reason for differentiating between the PBA and the PBI is that the use of the first is free while the use of the second is not^3^. In sum, we recommend the use of the PBI in studies aiming to compare burnout in two contexts, i.e., work and family, using a very common framework. The use of the PBA should be preferred in any other situation.

While we recommend the use of the PBA, it is not without limitations. The main limitation is that the PBA was created on the basis of the testimonies of burned-out *mothers* only. Although the congruence between factor scores of the two genders was acceptable and although the correlations with other variables were comparable across genders, mean burnout scores differed between genders: PBA scores were significantly lower for fathers than mothers. This gender difference was not found using the PBI. At this stage, we lack information to determine whether this result reflects higher sensitivity of the PBA to true gender differences or, on the contrary, a lack of sensitivity of the PBA to the detection of burned-out fathers. Future studies are urgently needed to go into this issue. These results also highlight the need for testimonies from burned-out fathers.

Another limitation is that the PBA was created on the basis of *French-speaking* and *English-speaking* samples only. The results show that the experience of burnout seems identical among Belgian, French, British, and American parents. Yet, this does not preclude the possibility that the structure and expression of parental burnout varies across countries or cultures. The countries surveyed here are very close to each other on many cultural dimensions (independent-interdependent self-construal, individualism-collectivism, gender roles, temporal orientation, etc.) and it is possible that the results would have been different if the study had been conducted in a country with opposite cultural characteristics and values.

These limitations leave plenty of room for future research. The most urgent direction stemming from the current research is the need to go deeper into the experience of burned-out fathers. A second, related, future direction is the need to examine cross-cultural variations in the expression and structure of parental burnout. A third pressing research direction concerns the need for clinical cutoff scores for the PBA and the PBI. In order to be valid, these clinical cutoffs must be set according to objective external criteria and not according to an arbitrary criterion such as the one used here to analyze the convergence between parental burnout instruments in detecting high levels of burnout (i.e., 66.6% of symptoms every day). Future studies using objective external criteria are therefore needed. The resulting clinical cut-off scores would allow epidemiological studies to determine the prevalence of parental burnout (i.e., the proportion of parents affected in a given location at a particular time), which is essential to both public health policy decision-making (highly prevalent disorders warrant preventive actions as well as training and staffing more specialized health professionals) and to clinical decision-making (if parental burnout is more common than myalgic encephalomyelitis, this is useful information in evaluating a stay-at-home mother describing intense fatigue).

## Ethics statement

This study was carried out in accordance with the recommendations of APA Ethical Principles of Psychologists and Code of Conduct with written informed consent from all subjects. All subjects gave written informed consent in accordance with the Declaration of Helsinki. The protocol was approved by the Psychological Sciences Research Institute, Université catholique de Louvain, Louvain-la-Neuve, Belgium.

## Author contributions

IR, MEB and MM designed the project and lead the data collection. IR computed the analyses, wrote the Method, and Results sections. MM wrote the Introduction and Discussion sections.

### Conflict of interest statement

The authors declare that the research was conducted in the absence of any commercial or financial relationships that could be construed as a potential conflict of interest.

## Footnotes

^1^Items EE1 to EE8 and PA1 to PA6 Copyright © 1981 Christina Maslach & Susan E. Jackson. All rights reserved in all media. Published by Mind Garden, Inc., www.mindgarden.com. Altered with permission of the publisher.

^2^Skewness and kurtosis indicated that 10 of the 23 final items displayed deviations from normality. Conceptually, these deviations from normality make sense: burnout is not expected to be normally distributed in the population. Like most mental health indicators, burnout is expected to present an asymmetric distribution (i.e., to be positively skewed, like most psychological disorders). However, as normality is a critical assumption underlying the maximum likelihood procedure used for CFA, log transformations of these items were computed and ensured a normal distribution. Then CFA was performed twice, once including transformed items and once including original items. Estimates and model fit indices were strictly similar. Therefore, only the results obtained from the analyses computed on original variables are presented.

^3^Copyright © 1981 Christina Maslach & Susan E. Jackson. All rights reserved in all media. Published by Mind Garden, Inc., www.mindgarden.com
