# Supplementary material for: A Step Forward in the Conceptualization and Measurement of Parental Burnout: The Parental Burnout Assessment (PBA)
Source: Front Psychol. 2018 Jun 6;9:758. doi: 10.3389/fpsyg.2018.00758 (PMC5998056; doi:10.3389/fpsyg.2018.00758)
Supplement: Supplementary file 1 [file Table_1.docx]

*S1* Loading parameter estimates in EFA for the 50 initial items

|  | Factor 1 | Factor 2 | Factor 3 | Factor 4 | Factor 5 | Factor 6 |
| --- | --- | --- | --- | --- | --- | --- |
| I’m very irritable in my relationship with my child(ren) | .189 | .119 | .121 | .410 | .440 | .164 |
| I’m so tired out by my role as a parent that sleeping doesn’t seem like enough | .039 | .048 | .331 | .690 | .153 | -.092 |
| I feel completely overwhelmed as a parent | .312 | .135 | .232 | .711 | .130 | -.036 |
| I feel exasperated by my child(ren) | .256 | .134 | .212 | .488 | .429 | .207 |
| I pay less attention to my children’s feelings | .350 | .178 | .072 | .222 | .549 | -.018 |
| Being a parent seems much harder than I imagined | .180 | .202 | .310 | .515 | .066 | .127 |
| I feel resentful of my child(ren) | .426 | .152 | .078 | .146 | .331 | .029 |
| I feel guilty as a parent | .021 | .435 | .215 | .460 | .139 | .244 |
| I feel as though I’ve lost my direction as a dad/mum | .268 | .546 | .204 | .489 | .166 | .212 |
| I only half-listen to what my child(ren) tell(s) me | .104 | .219 | .246 | .185 | .577 | .040 |
| I feel completely run down by my role as a parent | .261 | .166 | .438 | .711 | .120 | .039 |
| I have zero energy for looking after my child(ren) | .301 | .286 | .375 | .573 | .212 | -.116 |
| I no longer feel fulfilled as a parent | .482 | .447 | .203 | .297 | .256 | .103 |
| I feel frustrated in my role as a parent | .310 | .356 | .330 | .415 | .293 | .324 |
| I don’t think I’m the good father/mother that I used to be to my child(ren) | .197 | .642 | .187 | .341 | .208 | .163 |
| I’m becoming less and less involved in my relationship with my child(ren) | .257 | .511 | .134 | .077 | .437 | .069 |
| I feel as though being a parent takes too much effort | .437 | .186 | .380 | .261 | .216 | .103 |
| When I interact with my child(ren). I no longer have the energy to take a step back | .436 | .253 | .265 | .322 | .353 | .023 |
| I can’t stand my role as father/mother any more | .787 | .193 | .118 | .169 | .115 | .015 |
| I feel tense when I’m with my child(ren) | .494 | .319 | .255 | .295 | .350 | .241 |
| I feel like I can’t take any more as a parent | .732 | .216 | .102 | .306 | .232 | .130 |
| I feel alienated in my role as a parent | .521 | .225 | .278 | .429 | .105 | .183 |
| I sometimes have the impression that I’m looking after my child(ren) on autopilot | .197 | .262 | .465 | .340 | .333 | -.037 |
| I have the sense that I’m really worn out as a parent | .165 | .256 | .599 | .529 | .107 | .106 |
| When I get up in the morning and have to face another day with my child(ren),  I feel exhausted before I’ve even started | .245 | .215 | .628 | .418 | .091 | -.043 |
| I don’t enjoy being with my child(ren) | .539 | .335 | .298 | .074 | .157 | .144 |
| I feel like I can’t cope as a parent | .635 | .337 | .287 | .364 | .077 | .208 |
| My child(ren) is/are a source of anxiety | .239 | .289 | .504 | .136 | .249 | .382 |
| I tell myself that I’m no longer the parent I used to be | .268 | .686 | .254 | .219 | .228 | .133 |
| I do what I’m supposed to do for my child(ren). but nothing more | .427 | .370 | .363 | -.003 | .300 | -.148 |
| I feel like I’ve really had enough after spending time with my child(ren) | .552 | .145 | .400 | .155 | .360 | -.002 |
| My role as a parent uses up all my resources | .305 | .149 | .614 | .316 | .214 | .083 |
| I don’t seem to enjoy being a parent in the way that I was expecting | .470 | .437 | .404 | .162 | .177 | .082 |
| I can’t take being a parent any more | .704 | .334 | .190 | .142 | .026 | .014 |
| I wish I could take a day off parenting | .293 | .209 | .543 | .318 | .153 | -.017 |
| Because of my role as a parent. I’m no longer the person I used to be | .158 | .308 | .534 | .268 | .115 | .144 |
| I’m ashamed of the parent that I’ve become | .270 | .768 | .212 | .209 | .120 | .060 |
| My role as a parent weighs heavily on me | .149 | .360 | .659 | .246 | .053 | .149 |
| I’m no longer proud of myself as a parent | .242 | .783 | .255 | .213 | .096 | -.052 |
| I have the impression that I’m not myself any more when I’m interacting with my child(ren) | .331 | .650 | .313 | .184 | .221 | .067 |
| I’m no longer able to show my child(ren) how much I love them | .403 | .464 | .161 | -.055 | .296 | -.118 |
| I find it exhausting just thinking of everything I have to do for my child(ren) | .166 | .179 | .643 | .322 | .254 | .098 |
| Thinking of everything I have to do as a mum/dad makes me feel like staying in bed | .214 | .260 | .580 | .377 | .148 | -.100 |
| I’ve run out of patience in my relationship with my child(ren) | .483 | .246 | .321 | .278 | .368 | .016 |
| Outside the usual routines (lifts in the car. bedtime. meals),  I’m no longer able to make an effort for my child(ren) | .523 | .399 | .282 | .092 | .414 | -.173 |
| I feel as though my role as a parent leaves me empty inside | .620 | .312 | .323 | .187 | .177 | -.137 |
| I’m ashamed of myself as a parent | .304 | .782 | .237 | .180 | .068 | -.034 |
| I have the sense that I’m no longer myself as a parent | .380 | .496 | .485 | .166 | .111 | -.079 |
| I’m becoming less and less involved in parenting my child(ren) | .350 | .491 | .206 | -.074 | .369 | -.035 |
| I’m in survival mode in my role as a parent | .408 | .288 | .524 | .292 | .119 | -.132 |

*Note* EX: Exhaustion; ED: Emotional Distancing; FU: Feelings of being fed up; CO: Contrast in parental self
