# Supplementary material for: A Step Forward in the Conceptualization and Measurement of Parental Burnout: The Parental Burnout Assessment (PBA)
Source: Front Psychol. 2018 Jun 6;9:758. doi: 10.3389/fpsyg.2018.00758 (PMC5998056; doi:10.3389/fpsyg.2018.00758)
Supplement: Supplementary file 2 [file Table_2.docx]

*S2* Loading Parameter Estimates in EFA from the Four-Factor Solution for the 23-item Version of the PBA according to sample and gender

|  | French-speaking parents | | | | English-speaking parents | | | | Mothers | | | | Fathers | | | |
| --- | --- | --- | --- | --- | --- | --- | --- | --- | --- | --- | --- | --- | --- | --- | --- | --- |
|  | F1 | F2 | F3 | F4 | F1 | F2 | F3 | F4 | F1 | F2 | F3 | F4 | F1 | F2 | F3 | F4 |
| EX1 | .237 | .756 | .324 | .200 | .782 | .336 | .238 | .026 | .831 | .260 | .289 | .039 | .581 | .402 | .098 | .153 |
| EX2 | .449 | .493 | .597 | .215 | .813 | .264 | .204 | .182 | .765 | .351 | .226 | .137 | .835 | .165 | .153 | .080 |
| EX3 | .160 | .715 | .008 | .082 | .757 | .208 | .101 | -.001 | .735 | .179 | .110 | .048 | .639 | .169 | -.013 | .041 |
| EX4 | .294 | .520 | .365 | .333 | .714 | .188 | .223 | .300 | .670 | .255 | .226 | .341 | .746 | .092 | .073 | .172 |
| EX5 | .225 | .403 | .205 | .441 | .703 | .190 | .168 | .272 | .633 | .204 | .193 | .326 | .680 | .087 | .343 | .076 |
| EX6 | .264 | .637 | .343 | .315 | .641 | .334 | .238 | .220 | .642 | .322 | .269 | .237 | .615 | .211 | .249 | .272 |
| EX7 | .265 | .623 | .382 | .346 | .671 | .129 | .252 | .325 | .647 | .193 | .284 | .336 | .777 | .143 | .155 | .156 |
| EX8 | .299 | .416 | .136 | .453 | .583 | .280 | .140 | .393 | .508 | .292 | .189 | .425 | .602 | .226 | .313 | .107 |
| EX9 | .263 | .561 | .396 | .383 | .455 | .301 | .331 | .406 | .501 | .311 | .372 | .385 | .388 | .203 | .319 | .227 |
| CO1 | .778 | .347 | .189 | .119 | .341 | .703 | .221 | .207 | .354 | .750 | .202 | .146 | .350 | .567 | .174 | .256 |
| CO2 | .774 | .272 | .304 | .197 | .307 | .668 | .230 | .320 | .332 | .721 | .275 | .245 | .200 | .541 | .279 | .114 |
| CO3 | .790 | .229 | .232 | .288 | .237 | .733 | .314 | .300 | .266 | .735 | .335 | .248 | .119 | .881 | .295 | .143 |
| CO4 | .768 | .209 | .221 | .272 | .270 | .723 | .264 | .352 | .265 | .730 | .287 | .311 | .172 | .789 | .344 | .156 |
| CO5 | .692 | .270 | .257 | .364 | .345 | .561 | .301 | .371 | .322 | .623 | .323 | .353 | .335 | .385 | .402 | .216 |
| CO6 | .566 | .417 | .204 | .301 | .487 | .637 | .271 | .106 | .516 | .602 | .266 | .088 | .312 | .691 | .212 | .238 |
| FU1 | .399 | .395 | .690 | .265 | .175 | .218 | .763 | .206 | .214 | .238 | .792 | .185 | .228 | .233 | .252 | .809 |
| FU2 | .466 | .238 | .618 | .331 | .198 | .248 | .774 | .272 | .221 | .299 | .744 | .239 | .214 | .298 | .306 | .795 |
| FU3 | .405 | .570 | .326 | .350 | .401 | .318 | .658 | .186 | .377 | .240 | .668 | .217 | .223 | .254 | .563 | .384 |
| FU4 | .434 | .446 | .539 | .355 | .451 | .398 | .525 | .208 | .455 | .364 | .590 | .203 | .309 | .294 | .537 | .392 |
| FU5 | .470 | .172 | .465 | .507 | .271 | .264 | .497 | .377 | .273 | .324 | .523 | .337 | .225 | .307 | .589 | .310 |
| ED1 | .200 | .176 | .178 | .547 | .213 | .246 | .248 | .595 | .229 | .271 | .275 | .527 | .182 | .228 | .534 | .119 |
| ED2 | .376 | .335 | .189 | .648 | .284 | .291 | .227 | .710 | .239 | .298 | .372 | .622 | .234 | .156 | .811 | .107 |
| ED3 | .482 | .102 | .201 | .370 | .084 | .348 | .330 | .549 | .113 | .398 | .387 | .405 | -.025 | .261 | .716 | .101 |

*Note* EX: Exhaustion in parental role; CO: Contrast in parental self; FU: Feelings of being fed up; ED: Emotional Distancing.
